# Proprioceptive feedback modulates coordinating information in a system of segmentally distributed microcircuits

**DOI:** 10.1152/jn.00321.2014

**Published:** 2014-09-03

**Authors:** Brian Mulloney, Carmen Smarandache-Wellmann, Cynthia Weller, Wendy M. Hall, Ralph A. DiCaprio

**Affiliations:** ^1^Department of Neurobiology, Physiology, and Behavior, University of California, Davis, California;; ^2^Emmy Noether Group, Zoological Institute, University of Cologne, Cologne, Germany; and; ^3^Department of Biological Sciences, Ohio University, Athens, Ohio

**Keywords:** locomotion, proprioceptor, efference copy, coordination, burst strength

## Abstract

The system of modular neural circuits that controls crustacean swimmerets drives a metachronal sequence of power-stroke (PS, retraction) and return-stroke (RS, protraction) movements that propels the animal forward efficiently. These neural modules are synchronized by an intersegmental coordinating circuit that imposes characteristic phase differences between these modules. Using a semi-intact preparation that left one swimmeret attached to an otherwise isolated central nervous system (CNS) of the crayfish, *Pacifastacus leniusculus*, we investigated how the rhythmic activity of this system responded to imposed movements. We recorded extracellularly from the PS and RS nerves that innervated the attached limb and from coordinating axons that encode efference copies of the periodic bursts in PS and RS axons. Simultaneously, we recorded from homologous nerves in more anterior and posterior segments. Maintained retractions did not affect cycle period but promptly weakened PS bursts, strengthened RS bursts, and caused corresponding changes in the strength and timing of efference copies in the module's coordinating axons. Changes in these efference copies then caused changes in the phase and duration, but not the strength, of PS bursts in modules controlling neighboring swimmerets. These changes were promptly reversed when the limb was released. Each swimmeret is innervated by two nonspiking stretch receptors (NSSRs) that depolarize when the limb is retracted. Voltage clamp of an NSSR changed the durations and strengths of bursts in PS and RS axons innervating the same limb and caused corresponding changes in the efference copies of this motor output.

the nervous systems of arthropods and most terrestrial vertebrates contain dedicated neural circuits that control movements of individual limbs distributed in different segments of the central nervous system (CNS). Effective locomotion requires that these circuits be synchronized and coordinated and that this coordination be sensitive to perturbations of movements of individual limbs. The interplay of central coordinating circuits and proprioceptive feedback to individual limbs during locomotion is dynamic, and the extent to which coordination depends on proprioceptive feedback from each limb in each cycle of movements varies widely between different kinds of animals. This dependence is correlated with the accuracy required of each cycle of movement. Swimming and flying in fluid media, which do not require that the full weight of the body be supported by individual limbs during each cycle, require less accuracy than do, for example, climbing a blade of grass or galloping on rough ground.

The interplay of a central coordinating circuit and proprioceptive feedback can also reveal features of the CNS's operation. The crustacean swimmeret system has a comparatively low requirement for proprioceptive feedback, and fictive swimmeret beating can be recorded in the complete absence of feedback (Hughes and Wiersma 1960; Ikeda and Wiersma 1964). The complex motor pattern that drives each cycle of swimmeret movements involves synchronized output from four pairs of segmental microcircuits, and the intersegmental circuit that coordinates them is now known in cellular detail (Smarandache et al. 2009; Smarandache-Wellmann et al. 2014; Smarandache-Wellmann and Grätsch 2014). This circuit synchronizes oscillations of the microcircuits in different segments and imposes a posterior-to-anterior phase progression that is stable through a wide range of cycle periods. Key elements of this intersegmental circuit are coordinating neurons originating in each microcircuit that encode efference copies of each cycle of its output. How does proprioceptive feedback from individual swimmerets affect the output of this integrated system? At rest, the swimmerets of living prawns and crayfish are rotated anteriorly against the ventral surface of the next anterior abdominal segment (Fig. 1*A*). During forward swimming, each swimmeret periodically swings posteriorly (retraction) in a power stroke and then swings anteriorly (protraction) in a return stroke to its resting position, a periodic cycle of retraction and protraction.

**Fig. 1.**
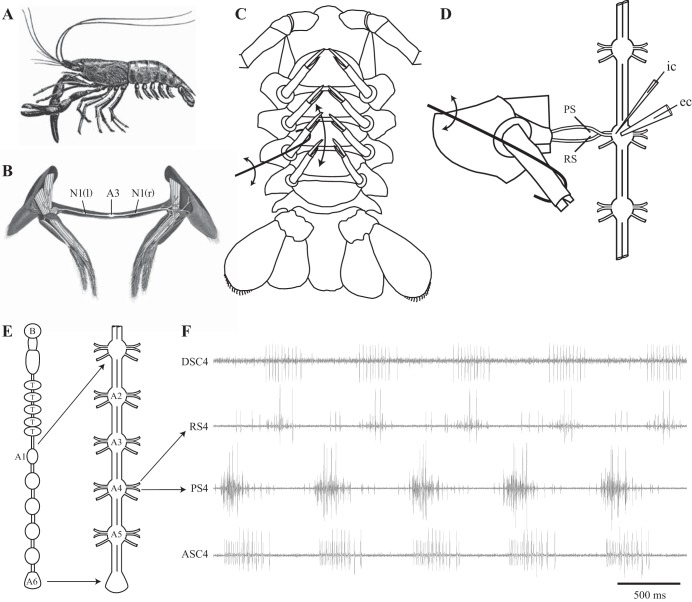
*A*: lateral view of a prawn that shows the pairs of swimmerets distributed on segments of the abdomen. From Huxley (1880). *B*: drawing of 1 pair of swimmerets that shows their attachments to the abdominal wall, intrinsic and extrinsic musculature, and the innervation of each swimmeret by the nerve (N1) that projects from the segmental ganglion (A3). From Keim (1915). *C*: drawing of the ventral side of a female crayfish abdomen that shows the 4 pairs of swimmerets in their resting, protracted positions. One swimmeret, on the right side of abdominal segment 4, has a hook attached to retract it posteriorly through the arc it makes during each cycle of power-stroke and return-stroke movements (double-headed arrow). *D*: drawing of the semi-intact preparation with 1 swimmeret still attached that shows the positions of the pin electrodes on the power-stroke (PS) and return-stroke (RS) branches of the N1 innervating the attached swimmeret. To permit swimmeret movements (double-headed arrow) and simultaneous recordings with a microelectrode (ic) in the lateral neuropil and a suction electrode (ec) on the minuscule tract, the N1 is rolled and the distal rami are pruned off. *E*: diagram of the crayfish central nervous system (CNS) showing the brain (B), the thoracic ganglia (T), and the chain of abdominal ganglia (A1–A6). The abdominal chain is expanded to show the segmental nerves that innervate swimmerets. *F*: simultaneous recordings from PS and RS branches of 1 N1 in ganglion A4 and from axons of ascending (ASC4) and descending (DSC4) coordinating neurons arising from the same hemiganglion.

Each swimmeret has its own four sets of sensory neurons (Mulloney and Smarandache-Wellmann 2012). Spiking afferents respond to deflection of setae on the distal rami of each swimmeret (Fig. 1*B*). Others respond to deformation of the cuticle of these rami (Killian and Page 1992a, 1992b). A third set of spiking afferents and a pair of nonspiking stretch receptors (NSSRs) (Heitler 1982) innervate sensory strands that span the joint between the coxa and the basipodite (CB joint) of each swimmeret (Davis 1969; Heitler 1986; MacMillan and Deller 1989; Miyan and Neil 1986). These strands are stretched by retraction or rotation of the swimmeret. The spiking afferents from these strands have peripheral cell bodies and thin axons that project through N1 into the segmental ganglion. The NSSRs, in contrast, have central cell bodies and large-diameter processes that extend from the ganglion's lateral neuropil (LN) (Skinner 1985b) through the anterior branch of N1 to reach the sensory strand. NSSRs are depolarized by stretching of their sensory strands that occurs during each retraction and conduct this graded depolarization as an analog signal from their peripheral terminals to their synaptic contacts within the LN (Heitler 1982, 1986).

To study the influence of proprioceptive afferents on the intersegmental coordinating circuit, we mechanically retracted one swimmeret into the position it would reach at the end of each power stroke and held it there while recording from the motor neurons and coordinating interneurons associated with that swimmeret (Fig. 1, *C* and *D*). Maintained retraction altered the timing and strengths of bursts of spikes in power-stroke (PS) and return-stroke (RS) motor axons that innervated the retracted limb. It also altered the timing and numbers of spikes fired in each cycle by the two coordinating neurons that encode efference copies of these PS and RS bursts. The changes in these efference copies affected the durations and phases of bursts of spikes in PS axons that innervated swimmerets in more anterior and more posterior segments but did not change the strengths of these bursts. The significance of these results and their relevance to earlier work on this system are discussed in the context of the recent description of the intersegmental coordinating circuit.

## MATERIALS AND METHODS

### 

#### Isolated nerve cord preparations.

Crayfish were first anesthetized on ice and then exsanguinated by replacing their hemolymph with crayfish saline. Then the ventral nerve cord from the fourth thoracic ganglion, T4, to the last abdominal ganglion, A6 (Fig. 1*E*), was removed to a Sylgard-lined dish filled with normal saline. Normal saline contained (in mM) 5.4 KCl, 2.6 MgCl_2_, 13.5 CaCl_2_, and 195 NaCl buffered with 10 Tris base and 4.7 maleic acid at pH 7.4. The sheaths were removed from the dorsal side of each ganglion. Isolated ventral nerve cord preparations sometimes spontaneously express the normal swimmeret motor pattern (Hughes and Wiersma 1960), but in other preparations that either were intermittently active or were silent a 1.5–3 μM solution of the cholinergic agonist carbachol (Sigma) in normal saline was superperfused over the preparation to elicit continuous expression of a stable motor output (Braun and Mulloney 1993; Chrachri and Neil 1993; Mulloney 1997).

#### Recording methods.

To record action potentials in the axons of PS and RS motor neurons, pin electrodes were placed on the posterior (PS) and anterior (RS) branches of the swimmeret nerves, N1, that project from each ganglion A2 through A5 (Fig. 1*E*) (Mulloney and Hall 2000). To record activity of the intersegmental coordinating neurons, ascending [ASC early (ASC_E_)] and descending (DSC), that originate in each module (Smarandache et al. 2009), we placed a suction electrode on the minuscule tract (MnT) as it crossed dorsal to the lateral giant axon (Mulloney et al. 2003; Skinner 1985a; Smarandache et al. 2009). All extracellular recordings were band-pass filtered and amplified with A-M Systems 1700 high-gain preamplifiers (Carlsborg, WA).

Intracellular recordings were made with npi SEC-05X amplifiers (npi electronic, Tamm, Germany) from processes of neurons within the LN with sharp glass microelectrodes. During each microelectrode experiment, neurons were tentatively identified by their physiological properties and then filled with an intracellular marker. This tentative identification was later confirmed or contradicted by the neuron's anatomy. Microelectrodes contained a solution of 1 M K^+^ acetate + 0.1 M KCl and 1% Dextran Texas Red (dTR) (Dextran Texas Red, mol wt 3000, lysine fixable; Life Technologies, Grand Island, NY). These electrodes had tip resistances of 30–50 MΩ. Neurons were filled iontophoretically with +1.0-nA current steps 0.25 s long at 2 Hz for 20 min.

Extracellular and intracellular recordings were digitized, usually at 10 kHz, with an Axon Instruments Digidata 1322A or 1440 and pCLAMP software (Molecular Devices, Union City CA) and saved as computer files for later analysis.

#### Semi-intact preparations.

To study the influence of a swimmeret's movements on the motor output to it and to other swimmerets, we developed a semi-intact preparation in which one or two swimmerets remained attached to the CNS (Fig. 1*D*). The ventral nerve cord (T4 through A6) was removed and pinned out dorsal side up as described above, except that the lateral abdominal wall and swimmeret of one segment, either segment 3 or segment 4, were left attached through N1 to ganglion A3 or A4. To allow the swimmeret to move, the piece of body wall was rolled 180° around the N1 and pinned securely to the Sylgard. The swimmeret itself was shortened by cutting off its distal rami so their movements would not hit the electrodes. The major PS and RS muscles of the swimmeret originate on the medial surface of the lateral abdominal wall (Fig. 1*B*), and in some of these preparations the swimmeret beat periodically in what appeared to be mechanically normal cycles of movements. Pin electrodes were placed on the anterior and posterior branches of the twisted N1 to record PS and RS spikes en passant (Fig. 1*D*). A suction electrode was placed on the MnT above the module innervating the attached swimmeret to record firing of coordinating neurons that originated in that module. In some experiments, a microelectrode was then brought in to record intracellularly from neurons in the module that innervated the attached swimmeret.

The outer end of the basipodite of the swimmeret itself was grasped by a hook fashioned from an insect pin attached to a micromanipulator or to the head stage of a puller controlled by a proportional-integro-differential controller (Aurora Scientific model 322C, Aurora, ON, Canada) operating in length-feedback mode. Commands to move the swimmeret using the controller were generated with a sine wave generator or with waveforms generated by pCLAMP (Molecular Devices).

#### Measuring parameters of the motor patterns.

**Fig. 2.**
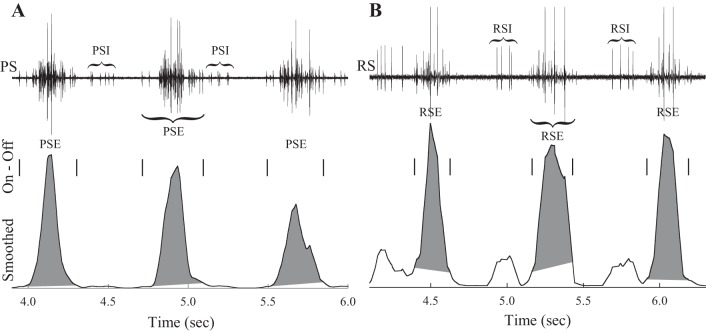
Calculating strengths of multiunit bursts of spikes in extracellular PS and RS recordings. *A*: 3 cycles of motor output recorded from the PS branch (Fig. 1, *E* and *F*). This recording includes both power-stroke excitor units (PSE) and power-stroke inhibitor units (PSI), whose bursts alternate. The times at which each PSE burst began and ended (On and Off) are marked with vertical bars. The smoothed, rectified PS recording is plotted beneath on the same time axis. The areas of the envelopes of the 3 smoothed PSE bursts between each burst's On and Off time are shown as gray polygons. *B*: 3 cycles of motor output recorded from the RS branch (Fig. 1, *E* and *F*). This recording includes both return-stroke excitor units (RSE) and return-stroke inhibitor units (RSI). The times at which each RSE burst began and ended are marked with vertical bars. The smoothed, rectified RS recording is plotted below, and the area of each RSE burst's envelope between its On and Off time is plotted as a gray polygon.

Each swimmeret is innervated by four types of motor neurons (Mulloney and Smarandache-Wellmann 2012) whose axons segregate into the anterior (RS) and posterior (PS) branches of each N1. About 35 return-stroke excitor (RSE) axons and 3 return-stroke inhibitor (RSI) axons run in the RS branch, while about 35 power-stroke excitor (PSE) axons and 2 power-stroke inhibitor (PSI) neurons run in the PS branch (Mulloney and Hall 1990, 2000). Neurons within each type fire simultaneously, but not all of these neurons reach threshold whenever the system is active. As excitation increases, neurons are recruited by increasing size; as it decreases, they drop out in the opposite order (Davis 1971). To distinguish bursts of spikes of different types in the same recording (e.g., Fig. 1*F*, Fig. 2), we used prior knowledge of the numbers of PSI and RSI units and their phase relations to PSE and RSE within cycles (Fig. 2). Recordings from experiments where RSE and RSI or PSE and PSI bursts could not be confidently distinguished were excluded from these analyses.

Digitized recordings were analyzed with Dataview (http://www.st-andrews.ac.uk/∼wjh/). The start times and stop times of bursts of spikes in each trace were marked with Dataview's cursor mode and used to describe the temporal structure of the periodic motor pattern. The times of PSE, PSI, RSE, and RSI bursts were marked separately (Fig. 2). The period of each cycle was the interval from the start of one PS burst, normally PS5, to the start of the next PS burst. The duration of each burst was the interval between the start and stop times. The phase of each burst within each cycle was calculated using the measured time from the start of the cycle to the start of the burst. Phase was then defined as the ratio of this interval to the cycle's period, and so could range from 0 to 1.0.

#### Measuring strengths of bursts of spikes.

In different cycles of motor output, the strengths of bursts of spikes in swimmeret motor neurons—the numbers of motor neurons recruited and the numbers of spikes each neuron fired (Davis 1971)—could vary. These strengths were measured with a modification of the method detailed in Mulloney (2005). First, the mean voltage was subtracted from each trace to remove any DC offset and the voltage at each time step was squared to rectify it. The rectified trace was then smoothed with a fast Fourier transform (FFT) with a triangular kernel. The half-width of this kernel was 327 time points, corresponding to 32.7 ms sampled at 10 kHz. The smoothed recording was restored with an inverse FFT. Then, using the lists of times at which each burst started and stopped, which had been measured independently with Dataview, we isolated the smoothed waveform of each burst and calculated its area (Fig. 2). Dividing each burst's area by its measured duration gave a measure of its strength that increased as the numbers and sizes of spikes within it increased, independent of changes in burst duration (Mulloney 2005). This procedure was effective even when an electrode recorded bursts of spikes in more than one functional group of motor axons (Fig. 2).

#### Statistical analysis.

In this report, an “experiment” means the manipulation of one swimmeret in one preparation. In those two preparations where two swimmerets were left attached on the left and right sides of one segment, each swimmeret was manipulated separately, the recordings were made with different electrode placements, and the data associated with the different recordings were analyzed as different, independent experiments. We did not pool data from different experiments. Each experiment could include a series of retractions and protractions or a series of voltage steps during voltage clamp of an NSSR. The analysis of each experiment pooled measurements of individual bursts from the same electrode recorded under the same conditions (for example, the series of retractions illustrated in Fig. 4). We calculated statistics of these measurements recorded under the same condition (for example, PSE durations during retractions and during protractions).

To test the probability that a parameter measured under two conditions during an individual experiment did not change, we calculated *t*-tests (Zar 1996), using the routines in SigmaPlot 12.5 (Systat, San Jose, CA). To test the probability that a parameter measured under two conditions did not change in a group of similar experiments, we calculated paired *t*-tests (Zar 1996), using SigmaPlot. Results of these calculations are reported as probabilities plus the power of each calculation.

#### Confocal microscopy and imaging procedures.

At the end of each experiment, the preparation was fixed overnight in 4% paraformaldehyde in phosphate-buffered saline (PBS). Preparations were then rinsed with PBS four times for 10 min each, pinned out dorsal side up, and cleared in an ascending ethanol series to methyl salicylate. Cleared whole mounts were mounted in methyl salicylate in a Permanox dish with a coverslip base. To prevent movement during imaging, a small, thick slip of glass was placed on top of the nerve cord.

Preparations were examined as whole mounts oriented for frontal view, dorsal side up (Fig. 1*E*). The structure of each labeled neuron was captured as a stack of confocal images that extended from the most dorsal to the most ventral part of the cell. Images were captured with an Olympus FluoView 300 confocal microscope (Olympus America, Center Valley, PA) equipped with krypton (488 nm) and argon (568 nm) lasers and an Olympus ×20 0.7 NA UPlanApo lens. Step size was 0.75 μm. The images were converted to 24-bit TIF images in FluoView software, where the gamma and intensity were adjusted to optimize the background intensity. The resulting images were then transferred to Adobe Photoshop for further adjustment of brightness, contrast, and sharpness. All images in each stack were adjusted uniformly.

## RESULTS

### 

#### Movements imposed on a swimmeret affect the motor output to that swimmeret.

**Fig. 3.**
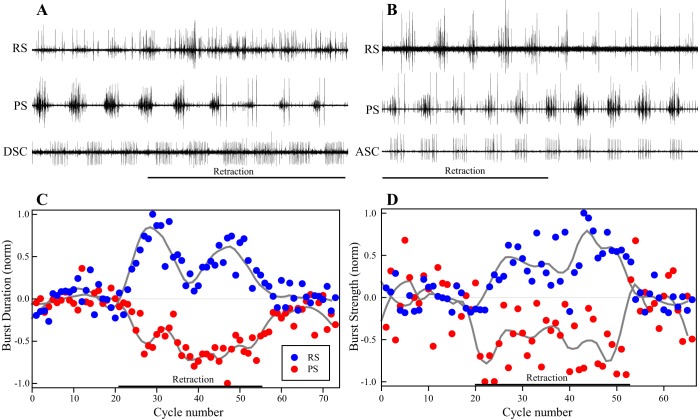
Changes in the motor output from a swimmeret microcircuit caused by mechanical retraction of its swimmeret. *A*: simultaneous recordings from PS and RS branches of the N1 that projects to the swimmeret and from the DSC coordinating axon that projects posteriorly from the module. The swimmeret was free to move for the first 4 cycles and then was held retracted (horizontal bar). The additional activity in the RS recording that began immediately upon retraction includes sensory afferents that had been silent. *B*: in a different preparation, simultaneous recordings from PS and RS branches of the N1 that projects to the swimmeret and from ASC axons that project anteriorly from the module. The swimmeret was held retracted during the first 5 cycles of activity (horizontal bar) and then released. The smaller spikes that begin each ASC burst are from the early ASC (ASC_E_) axon, while the larger spikes in each ASC burst during retraction are from the late ASC (ASC_L_) axon (Mulloney and Smarandache-Wellmann 2012). *C*: upon maintained retraction, durations of RS bursts increased while durations of PS bursts decreased. These differential changes in durations stopped once the swimmeret was released. *D*: retraction of the swimmeret also affected the strengths of PS and RS bursts. PS bursts were weaker and RS bursts were stronger during each retraction. The normal strengths of both PS and RS were restored once the retraction ended. In *C* and *D*, data have been normalized to the means of the bursts preceding the retraction, the thin horizontal line marks no change, and the gray lines are lowess fits to the experimental data.

**Fig. 4.**
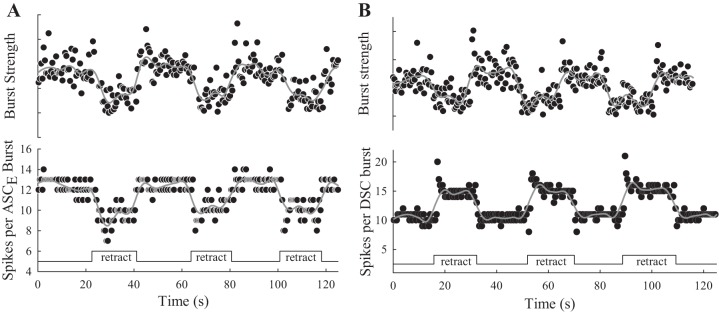
Differential responses of ASC_E_ and DSC axons to 3 imposed retractions (retract) of a swimmeret during continuous series of PS bursts (Fig. 1*F*, Fig. 3). *A*: during each retraction, PS bursts were weaker and the number of ASC_E_ spikes per burst decreased. The PS bursts that immediately followed the end of each retraction were transiently stronger than average. *B*: during each retraction, PS bursts were weaker and the numbers of DSC spikes per burst increased. DSC bursts immediately following the start of each retraction were transiently stronger than average.

In semi-intact preparations with an attached swimmeret that were actively expressing continuous, normally coordinated motor output, we retracted the swimmeret with a micromanipulator while recording from the PS and RS branches of the nerve innervating that swimmeret (Fig. 1*D*). Moving the limb from its resting protracted position to a fully retracted position immediately elicited a discharge of spiking sensory afferents in the RS branch (Heitler 1986; MacMillan and Deller 1989) and changed the durations and strengths of bursts of spikes in the PS and RS axons innervating the retracted swimmeret (Fig. 3*A*). When each retraction was halted and the limb returned to its fully protracted resting position, PS bursts and RS bursts promptly returned to the same levels they had before the retraction was imposed (Fig. 3*B*, Fig. 4). To quantify these changes, the parameters of a series of 12 or more bursts recorded during each retraction and protraction were measured. This sensory discharge in the RS branch complicated identification of RSE and RSI bursts (see materials and methods), but when a retraction was maintained these spiking afferents adapted after several cycles and fell silent (Heitler 1986). Therefore, the three cycles preceding each retraction or protraction and the first six cycles following it were omitted from quantitative analysis.

PS burst durations were shorter (2-tailed *t*-test: *P* < 0.001, Power ≥ 0.996) and RS burst durations were longer (2-tailed *t*-test: *P* < 0.001, Power = 1.0) during each retraction than those recorded before each retraction began. These same PS bursts were also weaker (2-tailed *t*-test: *P* ≤ 0.028, Power ≥ 0.606) and the same RS bursts were stronger (2-tailed *t*-test: *P* ≤ 0.025, Power ≥ 0.623) during each retraction than those recorded before the retraction began (*n* = 6 retractions in 2 experiments).

These maintained retractions did not affect the period of the system's motor output. During retraction, the mean periods were 1 ms shorter than when the limb was in its resting position (paired *t*-test: *P* = 0.437, *n* = 9 expts on 7 preps, Power = 0.052). In other experiments where the swimmeret was moved sinusoidally at periods close to the period of the preparation's expressed motor output, we did not observe entrainment of that output.

#### Imposed retractions also affected firing of coordinating neurons.

ASC_E_ neurons in each swimmeret module encode information about the timing, duration, and strength of each burst in the module's population of PS neurons. DSC neurons encode similar information about each burst in the population of RS neurons (Mulloney et al. 2006; Smarandache-Wellmann and Grätsch 2014). During each retraction of a swimmeret, bursts of spikes in coordinating axons originating in the module that innervated the swimmeret also changed (Fig. 3, *A* and *B*). The numbers of spikes per ASC_E_ burst decreased along with the decreased strengths of PS bursts (Fig. 4*A*; Table 1). At the same time, numbers of spikes per DSC burst increased as the strengths of PS bursts decreased (Fig. 4*B*; Table 1). In five experiments, ASC_E_ burst durations decreased during retractions while DSC burst durations increased (Table 1). The phases of both ASC_E_ and DSC bursts relative to PS4 also advanced (Table 1). These correlated responses of PS motor neurons and the ASC_E_ and DSC coordinating neurons are evidence that the proprioceptive afferents that respond to retraction and protraction of a swimmeret affect the module's pattern-generating kernel, not just the motor neurons themselves.

**Table 1.** Differences in burst parameters during retraction and protraction of a swimmeret

Neurons	Duration, ms	Strengths or Spikes per Burst*	Phase (relative to PS4)
PS3	−18.5 ± 10.7 (8)	0.077 ± 0.142 (8)	−0.049 ± 0.030 (7)
	*P* < 0.002	*P* = 0.166	*P* = 0.005
ASC_E_4	−72.3 ± 23.7 (5)	−2.85 ± 0.78 (5)	−0.029 ± 0.009 (5)
	*P* = 0.002	*P* = 0.001	*P* = 0.003
PS4	−64.6 ± 32.5 (8)	−0.996 ± 0.901 (7)	0
	*P* < 0.001	*P* = 0.017	
DSC4	84.2 ± 37.5 (5)	1.72 ± 1.65 (5)	−0.103 ± 0.043 (5)
	*P* = 0.008	*P* = 0.081	*P* = 0.006
PS5	21.1 ± 12.6 (5)	−0.024 ± 0.271 (5)	−0.062 ± 0.028 (4)
	*P* = 0.020	*P* = 0.850	*P* = 0.021

Data are mean ± SD differences (retract − protract) for no. of experiments in parentheses from experiments in which a swimmeret innervated by ganglion A4 was retracted.

* Power-stroke neuron (PS) statistics are burst strengths; ascending (ASC_E_) and descending (DSC) coordinating neuron statistics are numbers of spikes per burst. *P* = 2-tailed probability from paired *t*-test.

#### Retractions of one swimmeret also affected motor output to neighboring swimmerets.

Bursts of spikes in ASC_E_ and DSC neurons synchronize neighboring swimmeret modules (Smarandache-Wellmann et al. 2014; Zhang et al. 2014). Changes in the strengths or timing of these bursts affect the strengths and phases of the motor output from modules in neighboring segments (Mulloney and Hall 2007). Given this background, we compared the phases, durations, and strengths of PS bursts from more anterior and more posterior segments when a swimmeret in the segment between them was held in its resting protracted position and when it was held in its fully retracted position, thereby altering firing of the ASC_E_ and DSC neurons that originate in the retracted swimmeret's own module.

**Fig. 5.**
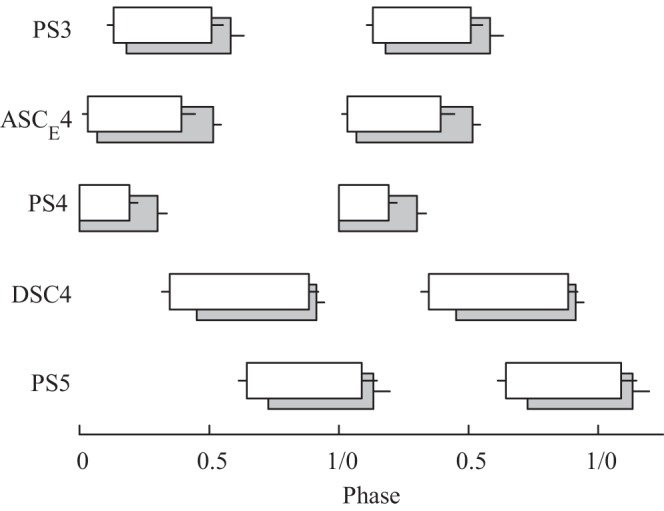
Box plots that compare phases and duty cycles of PS bursts in ganglia A3, A4, and A5 (PS3, PS4, and PS5) and of ASC_E_4 and DSC4 bursts recorded simultaneously on the same side of ganglion A4 during maintained protraction and retraction of a swimmeret. Gray boxes illustrate the mean duty cycles of bursts with the limb protracted, in its rest position. White boxes illustrate the mean duty cycles with the limb held fully retracted. Phases are defined within the cycle of PS4 bursts. Each box begins at the bursts' mean phase. Left-hand error bars show SD of phase; right-hand error bars show SD of duty cycle. The means of every parameter were different during retraction (*t*-test *P* < 0.001), except PS5 durations (*t* = −1.644, 338 df, *P* = 0.10).

The consequences of a maintained retraction for the PS motor neurons innervating the retracted swimmeret were different from the consequences for their homologs in neighboring segments (Fig. 1*E*, Fig. 5). Both durations and strengths of bursts in PS motor neurons innervating the retracted limb decreased significantly during retractions (Fig. 3; PS4 in Table 1). Simultaneously, durations of PS bursts in more anterior segments also decreased (PS3 in Table 1), while durations of PS bursts in more posterior segments increased (PS5 in Table 1). The phases of both anterior and posterior PS bursts were advanced during a retraction (Fig. 5; Table 1). However, in contrast to the strengths of bursts in PS motor neurons innervating the retracted limb (Fig. 3*D*), the strengths of PS bursts in these neighboring segments were unchanged (Table 1). Therefore, a retraction's major effect on neighboring modules was on the timing, not the strength, of their PS motor output.

#### Nonspiking stretch receptors.

**Fig. 6.**
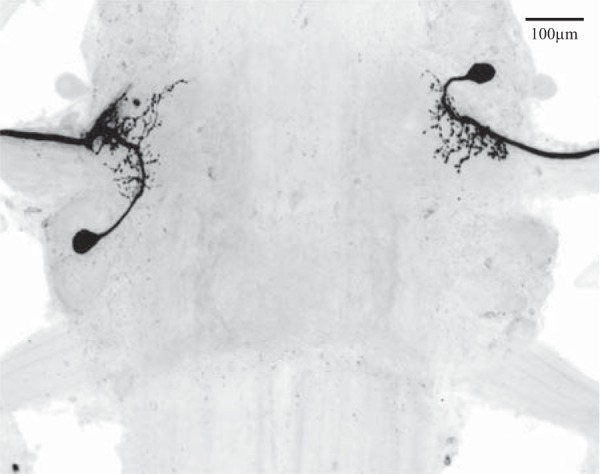
Whole mount of an abdominal ganglion, viewed from the dorsal side with anterior at *top*, in which 2 nonspiking stretch receptor (NSSR) neurons were filled with fluorescent dye. On *left*, an NSSR with a posterior cell body (NSSR-P) has small branches from its main process restricted to the left LN, and its axon projects into the left swimmeret nerve, N1. On *right*, an NSSR with an anterior cell body (NSSR-A) has small branches restricted to the right LN, and its axon projects into the right swimmeret nerve.

These results raise questions about proprioceptive feedback from a swimmeret to the module that innervates it. In two series of ablation experiments, Davis (1969) and MacMillan and Deller (1989) demonstrated that the sensory strands innervated by NSSRs are necessary and sufficient to elicit reflex responses to imposed swimmeret movements. We therefore focused our attention on these NSSR neurons (Fig. 6).

**Fig. 7.**
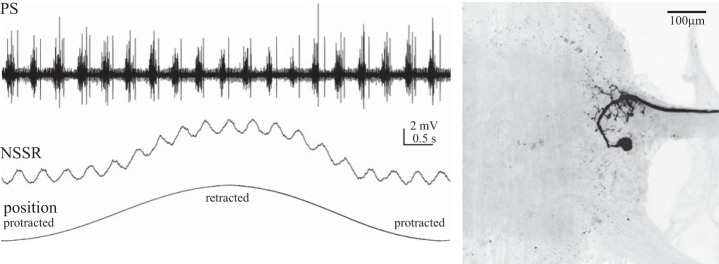
Intracellular recording from an NSSR, shown as a whole mount at *right*, within the LN during expression of normal swimmeret motor output and a slow retraction-protraction of the swimmeret through its full range of movement. The NSSR's membrane potential oscillates periodically in synchrony with the motor output (PS). These oscillations are superimposed on a larger depolarization caused by retraction of the swimmeret. The orientation of the whole mount is the same as that in Fig. 6.

When the system is actively expressing the normal swimmeret motor pattern, the membrane potentials of these receptors oscillate periodically in phase with the motor output because they receive synaptic input from some components of the module's pattern-generating kernel (Paul 1989). When a swimmeret was retracted in semi-intact preparations that were actively expressing normal motor patterns, recordings from the central processes of an NSSR innervating the retracted swimmeret showed a proportional depolarization superimposed on the periodic oscillations caused by periodic synaptic currents (Fig. 7). Retracting the NSSR through a wider angle caused a larger depolarization and greater inhibition of the PS motor output (Heitler 1982).

#### Depolarization of an individual NSSR altered its module's motor output.

**Fig. 8.**
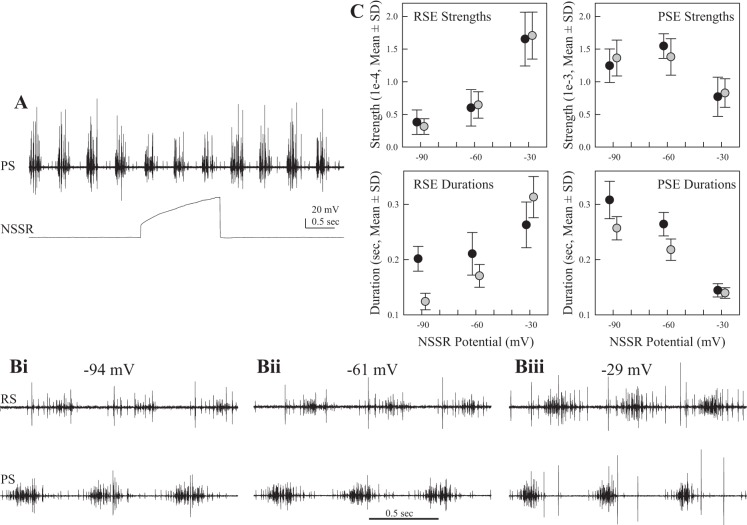
Responses of a module's motor output to changes in the membrane potential of 1 NSSR. *A*: depolarization of 1 NSSR with injected current partially inhibits periodic bursts in PS axons. *B*: simultaneous recordings of PS and RS activity during voltage clamp of an NSSR that innervated the same swimmeret. The neuron was clamped at −94 mV (*Bi*), 33 mV more hyperpolarized than its resting potential, −61 mV (*Bii*), and −29 mV (*Biii*), 33 mV more depolarized. *C*: plots of the durations and strengths of PSE and RSE bursts recorded in 2 experiments (black, gray) in which an NSSR was voltage-clamped at 3 different potentials. To avoid superposition, the symbols are offset slightly at each potential. As NSSR's membrane potential decreased, the durations and strengths of RSE bursts increased but those of PSE bursts decreased. In *Biii*, additional large PSI spikes were also recruited.

To explore the contributions of individual NSSR neurons to proprioceptive modulation of a swimmeret module's output, we made microelectrode recordings from NSSRs in the LN of isolated CNS preparations (Fig. 1*E*, Fig. 6) that were actively producing coordinated swimmeret motor patterns. We postulated that depolarization of an NSSR would cause changes in the module's output similar to those caused by retracting a swimmeret. Step depolarizations of an NSSR with current injections caused immediate weakening of PS bursts that promptly recovered when the step ended (Fig. 8*A*). Step changes in potential with discontinuous single-electrode voltage clamp (dSEVC) affected both PSE and RSE activity in the same module (Fig. 8*B*). Hyperpolarization to −94 mV, ∼30 mV below resting potential, increased durations of PSE bursts but shortened and weakened RSE bursts (Fig. 8*C*). Depolarization to −29 mV, ∼30 mV above resting potential, shortened and weakened PSE bursts but strengthened and lengthened RSE bursts (Fig. 8*C*). During these depolarizations, new larger RSE units and PSI units were recruited (Fig. 8*Biii*), as has been described in response to increases in excitation of the system (Davis 1971; Mulloney 1997).

Depolarizing an NSSR also affected coordinating neurons in the same module (Table 2). ASC_E_ bursts lost one spike per cycle, but their durations did not change significantly. DSC bursts, however, gained four spikes per cycle and lasted 80 ms longer (Table 2). The phases of both ASC_E_ and DSC bursts did not change during depolarization of the NSSR.

**Table 2.** Differences in burst parameters caused by depolarizing an NSSR neuron

Neurons in A4	Duration, ms	Spikes per Burst	Phase (relative to PS5)
ASC_E_ (*n* = 6 expts)	1.3 ± 12.3	−0.735 ± 0.933	0.0 ± 0.023
	*P* = 0.404	*P* = 0.056	*P* = 0.577
PS4 (*n* = 7 expts)	−42.8 ± 29.9	nd	0.029 ± 0.032
	*P* = 0.009		*P* = 0.028
DSC (*n* = 63 bursts in 1 expt)	79.9	4.4	0.042
	*P* < 0.001	*P* < 0.001	*P* = 0.199

Data are mean ± SD differences (depolarized − rest) from experiments where a nonspiking stretch receptor (NSSR) neuron in ganglion A4 was depolarized by current injection. *P* = 1-tailed probabilities from paired *t*-tests, except for *t*-test for unpaired DSC data. nd, Not determined.

Each swimmeret module has two NSSRs, NSSR-A and NSSR-P (Heitler 1982), that differ in the positions of their cell bodies (Fig. 6). In all of our experiments, we filled the recorded neuron and identified it as one of these types. Tabulating experiments by NSSR type failed to reveal any physiological difference between them, so the evidence is that they are functional equivalents operating in parallel.

#### How does this work?

Both depolarization of an NSSR and retraction of a swimmeret appeared to affect all the PS and RS motor neurons and both coordinating neurons within the same microcircuit similarly, so we think it is likely that these effects are caused by synaptic connections between the NSSRs and components of the microcircuit's pattern-generating kernel (Smarandache-Wellmann et al. 2013, 2014). One well-established pathway through which coordinating information from other modules tunes the strength and phase of a microcircuit's output is the connection between commissural interneuron 1 (ComInt 1) and the IRSh neuron in the microcircuit's pattern-generating kernel (Smarandache-Wellmann et al. 2014), but this is not the pathway through which NSSRs operate. Dual microelectrode recordings from ComInt 1 neurons and NSSRs in the same module revealed no evidence of connections between these neurons. Depolarizing an NSSR strongly enough to inhibit PS motor bursts completely did not affect ComInt 1's membrane potential (data not shown, *n* = 3 expts). Depolarizing or hyperpolarizing ComInt 1 strongly enough to affect the microcircuit's PS and RS motor output reduced the amplitude of NSSR's oscillations, but there was no evidence of time-locked postsynaptic responses (data not shown).

**Fig. 9.**
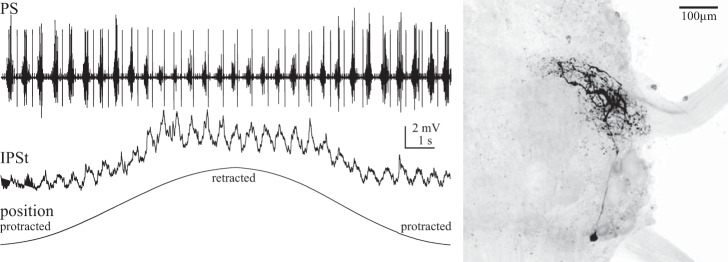
Simultaneous recordings of the membrane potential of an IPSt neuron, shown as a whole mount at *right*, and of PS activity in the same module during a cycle of retraction of the swimmeret. As the swimmeret was retracted, IPSt was depolarized and the PS motor neurons were increasingly inhibited. The orientation of the whole mount is the same as that in Fig. 6.

The kernel of each swimmeret module is composed of two classes of nonspiking local interneurons, IPS and IRS, that are connected by reciprocal inhibition to form the module's pattern-generating circuit. IPS and IRS inhibit PSE and RSE motor neurons, respectively (Mulloney 2003; Smarandache-Wellmann et al. 2013). During imposed retractions of a swimmeret, the membrane potential of one type of IPS neuron, IPSt, depolarized as the limb was retracted (Fig. 9). IPSt is a component of the microcircuit's pattern-generating kernel, so this observation is consistent with the idea that NSSRs synapse with the module's pattern-generating neurons.

## DISCUSSION

Retracting and holding a swimmeret retracted affected the periodic firing of PS and RS motor neurons in opposite ways. These responses would increase the strengths of contractions of return-stroke muscles that oppose the retraction and decrease the strengths of contractions of power-stroke muscles that assist the retraction. The two coordinating neurons, ASC_E_ and DSC, that originate in the microcircuit innervating the retracted limb were also affected in opposite ways (Table 1). The phases and numbers of spikes per burst in these neurons are efference copies of each PS and RS burst, and their opposite responses tracked changes in the timing and strengths of the different motor bursts. Maintained retraction also affected the timing of PS output both to more anterior and to more posterior swimmerets (Table 1), consistent with the altered strengths and timing of ASC_E_ and DSC bursts (Mulloney and Hall 2007).

### 

#### Each swimmeret's pair of NSSRs contributes to these responses to retraction.

Full retraction of a swimmeret should stretch all of the limb's position-sensitive afferents, and probably also stimulate some of its cuticular stress receptors. The sensory transduction apparatus of both NSSRs is inserted into an elastic strand that spans the swimmeret's CB joint and is stretched by retraction. Retraction of a swimmeret depolarizes the NSSRs that signal its position (Heitler 1982, 1983, 1986; MacMillan and Deller 1989; Miyan and Neil 1986). Although no detailed analysis of the transfer properties of swimmeret NSSRs is available, previous work indicates that the graded depolarizations of these afferents (Fig. 7) are relatively linearly proportional to receptor length, at least for 0.5- to 6-Hz sinusoidal movements (Heitler 1982). Rapid stretches produce graded depolarization that again tracks receptor length, plus an additional larger transient depolarization (Heitler 1982). These transient receptor potentials might underlie the transient responses to retraction that we see both in PS burst strengths and in numbers of spikes per burst in ASC_E_ and DSC neurons (Fig. 4).

We found that voltage-clamped depolarization of individual NSSRs simultaneously reduced PS firing somewhat and increased RS firing (Fig. 8*C*). Earlier ablation experiments indicated that the spiking afferents from setae on the distal rami of a swimmeret (Fig. 1*B*) played at most minor roles in modulating the force of swimmeret movements (Davis 1969; MacMillan and Deller 1989) but that sinusoidal currents injected into an NSSR could modulate firing of swimmeret motor neurons in the same microcircuit (Heitler 1986). Therefore, the physiology of individual NSSRs can account for some, but not all, features of a microcircuit's responses to retraction. Recall that each microcircuit has two NSSRs. Retraction should depolarize both NSSRs simultaneously, allowing them to act in parallel on their postsynaptic targets. By using a Vaseline-gap stimulation method that would depolarize both NSSRs simultaneously, MacMillan and Deller (1989) were able to entrain the system's output to the period of imposed voltage oscillations.

#### Failure of imposed movements to entrain the swimmeret motor pattern.

It is remarkable that the periodic alternation of PS and RS bursts to the retracted swimmeret did not halt during these maintained retractions (Fig. 3, Fig. 4); indeed, the period of the pattern did not change at all. This robust resistance to entrainment by movements of one swimmeret has been consistently reported in earlier studies (Heitler 1986; but see MacMillan and Deller 1989). In pioneering experiments that used command neurons (Acevedo et al. 1994; Wiersma and Ikeda 1964) to elicit swimmeret beating from preparations that had all swimmerets attached, West et al. (1979) found that mechanical interference with one swimmeret sometimes decreased the cycle period, but these effects were variable. In hindsight, this variability could be attributed both to variable manual retractions and to variability in the method used to elicit swimmeret beating. Only by moving several swimmerets simultaneously and synchronously were Deller and MacMillan (1989) able to entrain the system's output. Given that the system consists of four pairs of microcircuits, each with its own pattern-generating kernel, linked together by a coordinating circuit that distributes weighted information about each microcircuit's status to all the others (Smarandache et al. 2009), the robustness of period and relatively small changes in phase are understandable (Smarandache-Wellmann et al. 2014).

#### Comparisons with proprioceptive modulation during other forms of locomotion.

Because large-tailed crustaceans swim in a continuous fluid medium, and all but the largest are close to neutrally buoyant, forward swimming using the swimmeret system does not face some of the same mechanical problems that influence walking in terrestrial environments. During walking, both the load borne by each leg and the leg's position relative to the body are monitored by proprioceptors. These proprioceptors have major influences on each step, and on the coordination of these steps with those of other legs. In insects walking in different directions through an environment with different barriers, sensory feedback plays a crucial role in coordinating leg movements (Büschges et al. 2008). In stick insects, independent of the walking direction, the motor program switches from swing phase to stance phase when the leg's load sensors (campaniform sensilla) or position sensors (femoral chordotonal organ) are stimulated. When both sensory channels are activated simultaneously, a faster, more prolonged inhibition of the leg's flexor motor neurons is elicited (Akay and Büschges 2006). The role of NSSRs in the swimmeret system seems more like that of position sensors of the hip joint in walking mammals, which have major influences on each cycle of stepping. Prolonged retraction of a walking cat's hind leg alters the relative strengths of flexor and extensor motor output and also affects the timing of coordinated motor output to the other legs (Rossignol et al. 2006).

In the normal course of periodic swimmeret movements, the NSSRs of each swimmeret are depolarized only late in each power stroke (Heitler 1982), and the extent of each depolarization is determined by the angle of rotation reached at the end of the power stroke (MacMillan and Deller 1989). Similar nonspiking afferents in the walking legs of crabs achieve very high information transfer rates (DiCaprio 2004; DiCaprio et al. 2007), and graded transmission at other nonspiking neurons in these circuits increases sharply with increased depolarization (Smarandache-Wellmann and Grätsch 2014). Therefore, the NSSRs might add well-timed, proportional excitation that augments each RSE burst and the resulting return stroke, much as stretch receptors of locust wings contribute pulses of excitation that shape the centrally driven bursts of spikes in wing depressor motor neurons (Burrows 1975; Pearson and Wolf 1988) and influence the periods of wing beats during flight (Büschges and Pearson 1991; Möhl 1985; Wilson and Gettrup 1963).

In addition to locomotion, swimmerets are used by female crustaceans to carry fertilized eggs and newly hatched offspring (Gherardi 2002). During this protracted period of parental care, the female gently ventilates the attached egg masses by weak coordinated swimmeret movements with the same temporal structure as those seen during locomotion. However, the mechanical loads on each swimmeret are probably much greater, and proprioceptive feedback might well be more important in regulating these movements during these behaviors.

In summary, imposed retraction of one swimmeret has both local and distant effects on the ongoing activity of the swimmeret system. The local effects include weakening the drive to PS muscles and strengthening the drive to RS muscles as long as the retraction continues, both of which oppose the ongoing retraction. The local effects also change the timing and strengths of bursts of spikes in the local circuit's ASC_E_ and DSC neurons, which encode three parameters of each cycle: when each PS and RS burst began, how long it lasted, and how strong it was (Mulloney et al. 2006). We think these quantitative changes in ASC_E_ and DSC bursts—in the efference copies of each cycle—are decoded by their postsynaptic targets in other segments and cause their temporal responses to movement of one limb (Smarandache et al. 2009; Smarandache-Wellmann et al. 2014).

## GRANTS

This research has been supported by National Science Foundation (NSF) Grants 0905063 and 1147058 to B. Mulloney, National Institutes of Health (NIH) Grant NS-048068 to B. Mulloney, and a Burroughs Wellcome Collaborative Research Travel Grant to B. Mulloney and also by Emmy Noether DFG Grant SM 206/3-2 to C. Smarandache-Wellmann and a startup grant of the University of Cologne for female faculty to C. Smarandache-Wellmann. The confocal microscope used in this study was supported by NIH P30 EY-12576.

## DISCLOSURES

No conflicts of interest, financial or otherwise, are declared by the author(s).

## AUTHOR CONTRIBUTIONS

Author contributions: B.M. and R.A.D. conception and design of research; B.M., C.R.S.-W., W.M.H., and R.A.D. performed experiments; B.M., C.R.S.-W., C.W., and W.M.H. analyzed data; B.M., C.R.S.-W., and R.A.D. interpreted results of experiments; B.M. and C.W. prepared figures; B.M. drafted manuscript; B.M., C.R.S.-W., C.W., and R.A.D. edited and revised manuscript; B.M., C.R.S.-W., C.W., W.M.H., and R.A.D. approved final version of manuscript.
